# Supplementary figures and images for: Rapid molecular diagnosis of Parechovirus infection using the reverse transcription loop-mediated isothermal amplification technique
Source: PLoS One. 2021 Nov 29;16(11):e0260348. doi: 10.1371/journal.pone.0260348 (PMC8629174; doi:10.1371/journal.pone.0260348)

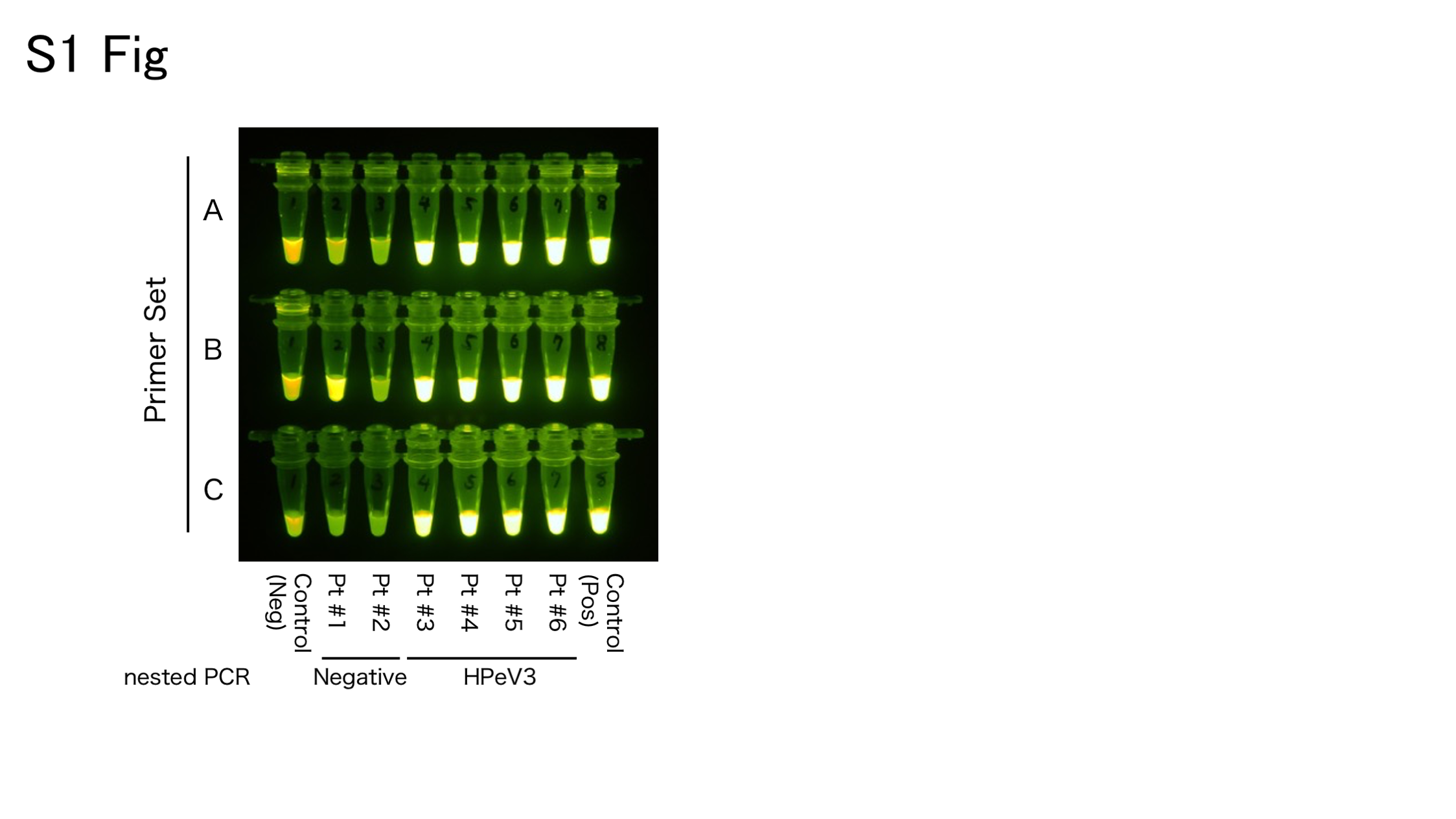

Supplement: S1 Fig — The RT-LAMP experiments were conducted using Sets A, B, and C. All sets show the same results. Neg, negative; Pos, positive; RT-LAMP, reverse transcription loop-mediated isothermal amplification. (TIF) [file pone.0260348.s001.tif]
