# Supplementary material for: Rapid molecular diagnosis of Parechovirus infection using the reverse transcription loop-mediated isothermal amplification technique
Source: PLoS One. 2021 Nov 29;16(11):e0260348. doi: 10.1371/journal.pone.0260348 (PMC8629174; doi:10.1371/journal.pone.0260348)
Supplement: S1 Table — (PDF) [file pone.0260348.s002.pdf]

S1 Table: Primer information for direct sequencing

	Sequence (5'→3')
Forward	AGCCATCCTCTAGTAAGT
Reverse	AGGCATCTGTTACCAGAT
