# Supplementary material for: Rapid molecular diagnosis of Parechovirus infection using the reverse transcription loop-mediated isothermal amplification technique
Source: PLoS One. 2021 Nov 29;16(11):e0260348. doi: 10.1371/journal.pone.0260348 (PMC8629174; doi:10.1371/journal.pone.0260348)
Supplement: S2 Table — (PDF) [file pone.0260348.s003.pdf]

S2 Table: The list of candidates of combinations of primers

Region in 5'UTR	Candidates of combinations of	Number of candidates							
		F1	F2	F3	B1	B2	B3	FIP	BIP
1-400	0	79	103	114	76	104	102	98	124
11-410	0	84	99	112	79	102	99	125	128
21-420	0	84	96	112	76	101	95	119	125
31-430	0	83	96	112	76	101	95	119	110
41-440	0	81	90	109	75	103	96	122	112
51-450	0	86	89	109	78	102	95	125	115
**61-460**	**1**	**87**	**90**	**110**	**78**	**106**	**97**	**125**	**115**
**71-470**	**2**	**86**	**88**	**107**	**75**	**102**	**94**	**119**	**115**
**81-480**	**3**	**82**	**90**	**107**	**72**	**103**	**95**	**122**	**122**
**91-490**	**3**	**80**	**91**	**106**	**73**	**104**	**95**	**122**	**127**
**101-500**	**3**	**79**	**92**	**106**	**73**	**103**	**95**	**127**	**124**
**111-510**	**3**	**79**	**92**	**106**	**70**	**103**	**95**	**127**	**119**
**121-520**	**3**	**79**	**92**	**106**	**70**	**103**	**95**	**127**	**119**
**131-530**	**3**	**79**	**92**	**106**	**67**	**101**	**95**	**127**	**119**
**141-540**	**3**	**71**	**85**	**103**	**60**	**96**	**93**	**118**	**106**
**151-550**	**3**	**65**	**85**	**100**	**55**	**97**	**88**	**117**	**99**
**161-560**	**3**	**58**	**87**	**107**	**48**	**98**	**89**	**108**	**104**
**171-570**	**3**	**62**	**88**	**108**	**52**	**100**	**91**	**108**	**105**
**181-580**	**3**	**69**	**85**	**103**	**54**	**98**	**88**	**108**	**104**
**191-590**	**3**	**67**	**81**	**97**	**52**	**92**	**82**	**108**	**102**
**201-600**	**3**	**66**	**82**	**97**	**54**	**94**	**82**	**108**	**108**
211-610	0	61	81	97	49	96	85	108	111
221-620	0	73	82	97	53	98	88	141	120
231-630	0	76	82	97	53	98	88	141	120
241-640	0	77	81	93	52	97	87	144	115
251-650	0	78	81	92	52	96	86	141	112
261-660	0	72	80	93	50	93	81	134	77
271-670	0	76	84	93	53	94	82	134	80
281-680	0	80	80	92	55	91	78	134	80
291-690	0	77	68	80	53	77	71	106	69
301-700	0	75	61	73	51	72	66	86	44
