# Supplementary material for: Rapid molecular diagnosis of Parechovirus infection using the reverse transcription loop-mediated isothermal amplification technique
Source: PLoS One. 2021 Nov 29;16(11):e0260348. doi: 10.1371/journal.pone.0260348 (PMC8629174; doi:10.1371/journal.pone.0260348)
Supplement: S4 Table — (PDF) [file pone.0260348.s005.pdf]

S4 Table: Result of the RT-LAMP changing reaction temperature

	Set A		Set B		Set C	
Temperature	WQ	HPeV3	WQ	HPeV3	WQ	HPeV3
59℃	(-)	(±)	(-)	(-)	(-)	(±)
61℃	(-)	(+)	(-)	(+)	(-)	(+)
63℃	(-)	(+)	(-)	(+)	(-)	(+)
65℃	(-)	(+)	(-)	(+)	(-)	(+)
67℃	(-)	(-)	(-)	(-)	(-)	(-)

214 ng of HPeV3 RNAs were applied to every tubes and reaction time of RT-LAMP was 60 min.
