# Supplementary material for: Rapid molecular diagnosis of Parechovirus infection using the reverse transcription loop-mediated isothermal amplification technique
Source: PLoS One. 2021 Nov 29;16(11):e0260348. doi: 10.1371/journal.pone.0260348 (PMC8629174; doi:10.1371/journal.pone.0260348)
Supplement: S5 Table — (PDF) [file pone.0260348.s006.pdf]

S5 Table: Result of the RT-LAMP changing RNA amount and reaction time

Virus	Origin	RNA amount (ng/tube)	Set A	Set B	Set C	PCR for enterovirus	PCR for adenovirus	PCR for HPeV
Adenovirus	Stool	254	Negative	Negative	Negative	***	***	***
Adenovirus serotype 1	Culture	114	Negative	Negative	Negative	Not tested	Positive	Negative
Coxsackievirus B5	Culture	82	Negative	Negative	Negative	**Positive**	Not tested	Negative
Echovirus 11	Culture	70	Negative	Negative	Negative	**Positive**	Not tested	Negative
Enterovirus D68	Culture	64	Negative	Negative	Negative	**Positive**	Not tested	Negative
Enterovirus A71	Culture	78	Negative	Negative	Negative	**Positive**	Not tested	Negative
Norovirus #1	Stool	148	Negative	Negative	Negative	***	***	***
Norovirus #2	Stool	296	Negative	Negative	Negative	***	***	***
Norovirus #3	Stool	222	Negative	Negative	Negative	***	***	***
HPeV1	Culture	62	**Positive**	**Positive**	**Positive**	Not tested	Not tested	**Positive**
HPeV3	Culture	70	**Positive**	**Positive**	**Positive**	Not tested	Not tested	**Positive**
HPeV3	Culture	88	**Positive**	**Positive**	**Positive**	Not tested	Not tested	**Positive**
